# Collaboration Between Researchers and Knowledge Users in Health Technology Assessment: A Qualitative Exploratory Study

**DOI:** 10.15171/ijhpm.2016.153

**Published:** 2016-12-16

**Authors:** Mylène Tantchou Dipankui

**Affiliations:** Department of Family Medicine, Université Laval, Quebec City, QC, Canada.

**Keywords:** Collaboration, Partnership, Integrated Knowledge Translation, Health Technology Assessment (HTA), Structuration Theory, Collective Impact Framework

## Abstract

**Background:** Collaboration between researchers and knowledge users is increasingly promoted because it could enhance more evidence-based decision-making and practice. These complex relationships differ in form, in the particular goals they are trying to achieve, and in whom they bring together. Although much is understood about why partnerships form, relatively little is known about how collaboration works: how the collaborative process is shaped through the partners’ interactions, especially in the field of health technology assessment (HTA)? This study aims at addressing this gap in the literature in the specific context of HTA.

**Methods:** We used a qualitative descriptive design for this exploratory study. Semi-structured interviews with three researchers and two decision-makers were conducted on the practices related to the collaboration. We also performed document analysis, observation of five team meetings, and informal discussion with the participants. We thematically analyzed data using the structuration theory and a collective impact framework.

**Results:** This study showed that three main contextual factors helped shape the collaboration between researchers and knowledge users: the use of concepts related to each field; the use of related expertise; and a lack of clearly defined roles in the project. Previous experiences with the topic of the research project and a partnership based on "a give and take" relationship emerged as factors of success of this collaboration.

**Conclusion:** By shedding light on the structuration of the collaboration between researchers and knowledge users, our findings open the door to a poorly documented field in the area of HTA, and additional studies that build on these early observations are welcome.

## Background

The collaboration between researchers and knowledge users in the health science field is increasingly promoted because it could enhance the effective use of research results in policy decisions and in practice settings.^1,2^ By involving decision-makers in the research process, it is suggested that it allows for their interaction with researchers around the tasks of asking and answering relevant questions^3^ for the common goal of solving complex health problems.^4^ Frequent interactions between the two groups are thought to assist researchers in developing research questions that are relevant to political priorities and contextual opportunities, and gain greater sensitivity to the decision-making context.^4,5^ Therefore, it is difficult for decision-makers to reject, discount, or ignore research results when they have contributed to them.^6^

For this reason, research funding agencies are emphasizing the importance of translating research results into practice and are experimenting with various strategies to achieve this outcome, including requiring knowledge users, such as practitioners or decision-makers, to become part of funded research teams.^7-9^ These partnerships are complex relationships^10^: they differ in form, in the particular goals they are trying to achieve, and in whom they bring together.^11^ Consequently, many have generated frustrations^12^ for failure to understand the dynamics.^10^ In addition, most collaboration initiatives are poorly and inconsistently described, evaluated, and reported, making it challenging to identify strong thematic areas.^13^ Although much is understood about why partnerships form, relatively little is known about how collaboration works^14^ - how the collaborative process is shaped through the partners’ interactions, especially in the field of health technology assessment (HTA). This study aims at addressing this gap in the literature.

In this article, the author describes the collaboration between researchers of the Quebec University Hospital Centre (QUHC) and Université Laval and knowledge users who are members of the HTA unit of the QUHC. This collaboration stemmed from a research project aiming to involve patients in the assessment of alternative measures to restraint and seclusion among adults in short-term hospital wards (in psychiatry) and long-term care facilities for the elderly,^15^ funded through a Canadian Institutes of Health Research (CIHR) Knowledge to Action (KTA) grant. Three patient involvement strategies were used: direct participation of patients’ representatives in the activities of the working group (including a selected committee) set up by the HTA Unit of the QUHC to assess alternative measures to restraint and seclusion; consultation with patients and their family members about their opinions and perceptions of seclusion and restraint and their alternatives by five focus groups held in short-term psychiatry care and three in long-term care facilities for the elderly; direct participation of patients’ representative in developing information material to present HTA report and recommendation to service users.

Particular attention is given to the author’s experience of being involved as a PhD student in the research team project, recruited to evaluate patient involvement in this HTA. The evaluation had three components: patient participation in the working group activities set up to assess this HTA^16^; the effects of patient involvement in this HTA^17^; and the collaboration between researchers and decision-makers. The present article focuses on the last component.

The aim of this study is to examine the impact of social context on the collaboration between researchers and knowledge users involved in this HTA and its facilitators.

## Methods

### Analytical Framework

The foundational collaborative literature is generated across several disciplines^6,10,18-23^ which illustrates that the factors that influence collaboration transcend contexts. The innovative element of this project is the use of the structuration theory and the collective impact (CI) framework as conceptual foundations.

The analytical framework draws on Giddens’ structuration theory to explain the impact of social context factors on collaboration, and on the CI framework to highlight factors of success of this collaboration. In fact, structuration theory^24,25^ provides a relevant analytical framework for understanding the actions of researchers and decision-makers by linking the structural elements to the context in which they are located. Giddens’ structuration theory describes institutions (ie, symbolic order/mode of discourse, legal, political, and economic institutions) as a set of rules and resources which stretch across time and space and are recursively implicated in the reproduction of social practices. These social institutions affect collaborative initiative and vice versa. Institutions are interrelated through three structures: Signification, domination, and legitimation. Signification (S) refers to structures of meaning or symbols based on representations that come from stocks of knowledge of actors and that are used in their communications; Domination (D) refers to structures of control and power that allows others to bend to one’s will using two types of resources: authoritative, which refers to the influence of some actors on others, and allocative, which refers to the control of physical aspects such as equipment or goods. Giddens talks of political institution when authoritative resources are used and economic institution when allocative resources are used. Finally, Legitimation (L) refers to structures conferring rights and obligations. These rights and obligations remind actors of what to do and the consequences of their doing so. Sanctions help to maintain and respect these formal codes. Therefore, the normative dimension refers to the rights and obligations that allow actors to justify their actions. These modalities are ranked S-D-L in symbolic institutions (orders/modes of discourse); D-S-L in political and economic institutions, and L-D-S in legal institutions***. ***According to Giddens,^24,25^ structures of signification always have to be grasped in connection with domination and legitimation; the three modalities are interrelated in practice.

The five elements of CI initiatives^26^ are also considered in the analytical framework: a common agenda (all participants have a shared vision for change including a common understanding of the problem and a joint approach to solving it through agreed upon actions), shared measurement systems (collecting data and measuring results consistently across all participants ensures efforts remain aligned and participants hold each other accountable), mutually reinforcing activities (participant activities must be differentiated while still being coordinated through a mutually reinforcing plan of action), continuous communication (consistent and open communication is needed across the many players to build trust, assure mutual objectives, and create common motivation), and backbone support organizations (creating and managing CI requires a separate organization(s) with staff and a specific set of skills to serve as the backbone for the entire initiative and coordinate participating organizations and agencies).^26^

### Study Design and Participants

A qualitative descriptive design using interviews (n = 5), observation (n = 5) and document analysis (n = 13 (3 publications and 10 presentations)) was chosen for this exploratory study. The author was a PhD student recruited by the research team to perform the evaluation of the patient involvement component of this HTA project. Before her recruitment, she knew three of the researchers, including her PhD supervisor, but did not know the other team members. A workshop was organized with researchers and knowledge users at the onset of this study in order to present the objectives and evaluation questions. Other meetings took place between the author and members of the team during the project for planning interviews to evaluate the interventions, data analysis and drafting of articles (see Table 1 for more details). The author performed interviews with a convenience sample of 5 of the 11 members of the research team. They were selected on the basis of their profession (researcher or knowledge user), the activities in which they had agreed to participate according to the task distribution table developed in this project, and their availability for an interview. Participants who were neither researchers nor knowledge users, not able to get involved in research project activities, or unavailable for an interview were excluded from the study. The interview guide, based on a conceptual framework, included five main questions aiming to gather information about the reasons and expectations of participants in relation with their collaboration, the characteristics of this relationship, the influence of this collaboration on the results of the research project, and the potential interest of respondents in other studies in which researchers and knowledge users would be involved.

**Table 1:** Task Distribution Table That Project Coordinator Used to Solicit Team Members

**Tasks to Be Performed**	**Team Members**											
	**PC**	**R1**	**KU3**	**KU2**	**KU1**	**R6**	**R2**	**R4**	**R5**	**R3**	**PhDS**
Project submission to the research ethics board	x	x									
**Objective 1. Validation of the Reference Framework of User Involvement in HTA**											
Prepare framework to validate	x	x				X	x		X	x	
Prepare interview guide	x	x							X		
Selection and recruitment for interviews	x										
Conducting interviews (about 20)	x										
Analyzing interviews	x	X	x							x	
Draft of the report or an article	x	x	x				X	x		x	
**Objective 2. Development of User Involvement Interventions**											
2.1. Patient participation in the evaluation process (multidisciplinary working group):											
Select patient community organization and contact the leaders	x	x		X	X	x					
Select 2 or 3 persons among those interested	x	x		X	X	x					
2.2. Consultation (data collection) among service users (from September 2011)											
Draft a list of community organizations to contact (users committee, associations, etc)	x			X	X				X		
Recruitment (about six focus groups)	x			X	X				X		
Finalize the interview guide	x	x		X	X				X		
Conduct focus groups	x			X	X		X-1-2		X		
Analyze focus group data	x		x	X	X						
Draft and integrate the report in the HTA report (planned for March 2012)	x	x	x	X	X		x	x			
2.3. Service user participation in the dissemination and communication of results (from April 2012)											
Produce a draft of information materials adapted to patients									X		
Organize focus groups with patient representatives – selection and recruitment (about 3)	x					x					
Conduct focus groups	x						X-1				
Produce a final version of information material to disseminate	x							x	X		
Disseminate results	x					x	x				
**Objective 3. Evaluation of Different Types of Patient Involvement Strategies**											
Workshop with researchers and knowledge users: drawing up of objectives and evaluation questions (fall 2011)	x	x	x	X	X		x		X	x	x
Interviews with various stakeholders to evaluate all the interventions											x
Estimation in terms of costs, resources, etc.				X	X						x
Analysis and drafting of articles		x					x	x			x

Abbreviations: HTA, health technology assessment; PC, project coordinator; R, researchers; KU, knowledge users; PhDS: PhD students.

The author contacted researchers and knowledge users involved in the activities of the project by email. Interested participants replied directly by email or face to face. The author sent a reminder email with a draft of the interview guide to all participants two days prior to the interview to confirm the time and location and if they were still available to participate.

### Data Collection Process

The author performed semi-structured interviews with participants, comprising standard open-ended questions supplemented by probes, during the month of July 2012. Audio recordings were made during four interviews and notes were taken during one at the request of the participant. The interviews, lasting an average of 30 minutes, took place face to face at the time and place that best suited participants. Interview content was transcribed verbatim and a summary of each interview was sent to the participant concerned to confirm their content.

Direct and unstructured observations of the five meetings of the research team, held on May 19, 2011, June 21, 2011, July 5, 2011, February 24, 2012, and April 20, 2012, were also carried out (See Table 2 for more details on meetings).

**Table 2:** Research Team Meeting Details

**Date**	**Participants**	**Purpose**	**Outcomes**
May 19, 2011	3 KU 1 Research agent of KU team 4 R 1 PhDS 1 PC	The approach to be taken to complete the project within a reasonable timeline	- Utilize the healthcare organisation’s internal resources to organise the focus groups; - Recruit the patients through a grouping of community organisations; - Present the project to network stakeholders in order to sensitize them to the study; - Study the effectiveness of alternative measures to restraint and seclusion, cost, efficiency; and organizational issues.
June 21, 2011	1 Research agent of KU team 1 PC 1 PhDS	Relevant methods for patient recruitment	- Recruit the patient representatives through the community organisations; - Work directly with the health facilities to identify participants for the focus groups.
July 5, 2011	4 R 1 PhDS 1 PC	Elements to consider in developing the reference framework of patient involvement	- The framework may include: Objectives - Who should be involved – For what technology – at which level; - Adapt the message to decision-makers; - Link the concepts to concrete examples.
February 24, 2012	1 R 1 Knowledge users 2 Research agent of KU team 1 PC 1 PhDS	Presentation of results of focus groups with patients	- Appreciation of work done; - Conclusions consistent with the study conducted with managers by HTA unit; - Highlight the critical points for the HTA report.
April 20, 2012	3 R 2 Knowledge users 1 PC 1 PhDS	The drafting of the assessment report in order to integrate the patient perspective	- Integrate the methodology of the two studies (Study conducted with managers and study conducted with patients); - Use the report on the study conducted with managers as guide to ensure that all the document is consistent.

Abbreviations: HTA, health technology assessment; PC, project coordinator; R, researchers; KU, knowledge users; PhDS: PhD students.

The starting up meeting of the project took place in May 2011, and involved nine persons including three knowledge users and a member of their team, four researchers, a PhD student, and the project coordinator. This meeting focused essentially on the approach to be taken to complete the project within a reasonable timeline so that the results have a quick impact on practices. The meeting lasted about two hours. Another meeting was held in June 2011 between the research agent (member of the HTA unit in charge of the project) and the project coordinator. This meeting lasted about 30 minutes and focused on the relevant methods for recruitment of patients.

The third meeting, between researchers only, took place in July 2011. This meeting focused on elements to consider in developing the reference framework of patients involvement in HTA. Four researchers, the PhD student and the project coordinator were present and the meeting lasted about 3 hours.

The fourth meeting was held in February 2012 and focused on the presentation of the results of focus groups with patients conducted by researchers and involving knowledge users. A researcher, a knowledge user and two members of his or her team were present, along with the project coordinator and the PhD student. The meeting lasted about 2 hours 30 minutes.

The fifth meeting was held in April 2012 and focused on the drafting of the assessment report in order to integrate the patient perspective and key issues to be addressed. Two knowledge users and three researchers, the project coordinator and the PhD student were present and the meeting lasted about 2 hours 30 minutes.

During each of these meeting, the author was both participant when giving her opinion about the topic discussed or when she had something to share, and an observer when taking notes on interaction (and discussions) between members of the team. The author was involved in all central activities of the project and the team members knew her role in this research. These observations aimed to capture the setting in which the team worked and their interaction in a physical environment (more natural circumstances). The field notes taken during these observations described essentially the process of activities and how participants behaved and interacted. The author also included personal thoughts about being in the field and her comments on participants’ views in these field notes.

### Data Analysis

The verbatim transcripts from the four interviews and field notes were analyzed using NVivo 8 software. A thematic analysis^27^ was performed according to the method described by Miles and Huberman^28,29^ comprising coding, organization, and linking. The author analyzed the five interviews with support from two people (her thesis supervisor and a research agent with expertise in qualitative analysis). Firstly, the author and the research agent coded independently. They compared their codes and differences in coding were resolved with discussions with a third person (the thesis supervisor) to ensure the reliability of the results. Secondly, the author coded the remaining interviews following the list of themes and sub-themes previously elaborated from the analysis of the first two interviews. The codes were labelled using short phrases taken from the participants’ own words. The author then sorted the different codes into potential themes and collated all the relevant coded data extracts within the identified themes and sub-themes. As analysis progressed, the codes, themes and sub-themes were revised and redefined. Finally, the author and the research agent together reviewed some codes and extracts for which doubts existed with regards to their codification.

The author analysed three published papers and ten presentations and their abstracts related to the research project. She also examined emails from May 2011 to October 2013 to study the interaction between members of the team. The analysis took place in three steps: (1) The author identified the research team members listed on the article, the abstract or the presentation; (2) The author then checked in the table of task distribution of project the role that each of them was engaged to play in the project; (3) The author then validated through emails their real contribution. The information was consigned in field notes.

The author used field notes^30^ including notes from unstructured observation of team meetings, inserted in the NVivo program in the form of memos to validate and complete the information gathered during the interviews. Finally, the author linked the different themes and sub-themes to the analytical framework for analytic generalization. A first draft of data analysis was sent to two researchers and a decision-maker in order to elicit their feedback on the content.

## Results

### Participants

A total of nine participants (six researchers and three knowledge users) appeared on the grant application and could participate in the study. However, four members of the team (three researchers and a knowledge user) were not approached: one researcher because he never attended team meetings, and three others because of their unavailability during the study period. Finally, three researchers and two knowledge users were interviewed. Concerning the impact of social context on the collaboration, five main themes related to institutions emerged from analysis: (1) The use of concepts related to each field that makes communication difficult; (2) Use expertise and the context to do research to maintain one’s contribution essential to the project; (3) Organizational constraints that require constant adjustments; (4) An office design leading to unequal access to the information; (5) Lack of knowledge concerning what the exact roles when collaborating should be. Previous experiences with the topic of the research project and a partnership based on “a give and take” relationship emerged as factors of success of this collaboration.

In the next sections, findings are presented according to the main themes in relation with the analytical framework.

The Impact of Social Context on the Collaboration Between Researchers and Knowledge Users

### The Symbolic Order/Mode of Discourse

#### The Use of Concepts Related to Each Field That Makes Communication difficult

Participants talked about difficulties to understand concepts related to each field. Knowledge users talked about difficulties to understand theories discussed by researchers.

*“I do not always feel that we understand each other [researchers and decision-makers] because there is a reality on the side of researchers. […] with many theoretical foundations to support the approach. This is normal. And I feel that some decision-makers are on the edge of their seats; they get impatient because they have not grasped all of that”* [KU1].

Some researchers questioned the scientific aspect of the HTA because of unfamiliarity with the approaches.

*“…when we are with them [knowledge users], I try to use plain language.... Then they probably do the same thing. They likely use layman’s terms; this has happened on several occasions. […] But I would ask what they meant, that stage or this approach... Because I wasn’t at all familiar with any of it. […]”* [R2].

R2 wonders if knowledge users are researchers? A discussion raised about how they do research. Researchers think that they should engage researchers. […] because the component design of patient engagement strategies needs help from researchers. R2 finds that when decision-makers are speaking, when she hears the discussion, it’s two different perspectives. R1 wonders why the researchers and knowledge users carried out two separate literature reviews on the same topic (Field notes).

Finally, the analysis of field notes also revealed that during the first meeting of the working group, a knowledge user highlighted the need to develop “measurable indicators” throughout the research project to bring the theoretical concepts to practical application.

### The Political/Economic Institution

#### Use Expertise and the Context to Do Research to Maintain One’s Contribution Essential to the Project

Collaboration seems to have close links with power relations – the influence of some participants on the others through the control of resources. Participants highlighted the importance of the contribution of knowledge users in the research project and the researchers’ effective use of information. Knowledge users used their HTA expertise in their communications. They were especially concerned with technical and feasibility elements while researchers were more interested in methodological elements such as what framework to use to perform patient involvement strategies. Thus, field notes analysis revealed that the approach to be taken to perform patient involvement strategies within a reasonable time frame was discussed during the first meeting; knowledge users suggested the presentation of the research project in a regional meeting to sensitize health facilities stakeholders to the study. Knowledge users also offered the context to study patient involvement strategies through the activities around HTA of alternative measures to restraint and seclusion, as a knowledge user pointed out:

*“... in the project, there is need for HTA expertise. It fact, we lead the project […] basically, we offered the context to do the research. We made a contribution, too. […]”* [KU2].

A researcher (R2) noted the importance of having knowledge users participating in team meetings to precisely communicate the information required for the advancement of the project.

*“They attend meetings because they know they have information to give us. And we need that information. This [attending meetings] is easier than using email and so on. So it allows us to establish an exchange, a working group”* [R2].

Researchers, in turn, use the information in such a way that decision-makers generally have access to information that they need for the project to progress. To this end, the document analysis revealed the existence of a task distribution table (Table 1) that was sent to each of the team members to indicate the activities and phases of the project they wished to be involved with. This task distribution table served as a landmark for the project coordinator regarding people to solicit at various phases of the research project. Thus, outside of project meetings, knowledge users had less information about the contribution of researchers to other research activities (such as stages of patient recruitment, strategies adopted, follow up process, etc). In this sense, a knowledge user expressed some frustration:

*“In fact is X, XA, XB ... I feel their involvement… and their willingness to contribute so much. Of course there are a whole bunch of co-researchers ... who contribute less, shall we say, as I see it anyway. I do not know exactly what they are going to have to bring to the table...”* [KU2].

#### Organizational Constraints that Require Constant Adjustments

Participants also stressed organizational constraints affecting collaborative efforts. Researchers talked about delays in the notification of acceptance of the grant application while knowledge users mentioned the difficulties to change a process already underway.

*“I think we have been aware from the beginning of the project. One has kept us informed of developments. And then … can we still do better? Yes. Patient selection, perhaps ... participating in the working group, maybe we should have try to predict it earlier in time. But we were caught in the constraints when whoops, suddenly a funding agency decides to give the budget ... We can’t wait to moving ahead. The train was already moving…we know six months later that we will have the funds […] All in all, given the circumstances, we have to applaud. […] Can we do better next time? We will try. But under the circumstances, I think we have done things well […]”* [KU1].

Consequently, the absence of patient representatives at the first meeting of the working group represented a limitation to this project according to a researcher.

*“I would tell you that [relatively speaking, knowledge users have limited access to the field]. Sure, again, the constraints were a little out of their power. Because if they had been able to involve [patient representatives] at the first meeting of the working group, before the work started, it would have been much better. The fact that patients arrive like that, at the second meeting, is ... a major limitation”* [R1].

#### An Office Design Leading to Unequal Access to the Information

Two knowledge users and a researcher (KU1 and KU2, R2) pointed out office design to explain unequal access to the information. They talked about the informal meetings (outside of team meetings) between team members working in the same building versus those located elsewhere.

*“Because there are areas in which I find they [knowledge users] are more rigorous than I am, and sometimes, there are things I find I’m much more rigorous than they are [...]. But maybe, as I say, as X and HTA unit are in the same building, maybe there are more informal meetings that I’m not aware of”* [R2].

Finally, both groups stressed the importance to realize the project within a reasonable time frame so that the results have a quick impact on practices.

### The Legal Institution

#### Lack of Knowledge Concerning What the Exact Roles Should Be When Collaborating

Researchers considered that knowledge users were available to get involved in the writing of the grant application and for meetings. But practically their involvement was limited to their presence at team meetings and the expression of their views during meetings. A researcher stressed the lack of knowledge user involvement in the reading and analysis of documents: “So the fact that they are available to do so [be present at team meetings] allows us to carry out an exchange and form a working group. But I do not feel that they are there to get the job done” [R2].

This point of view was not totally shared by knowledge users. They seemed unaware of researchers’ expectations concerning their role in the research project. They viewed their involvement in the stages of the research project as consisting mostly of answering researchers’ requests.

*“I think so ... I do the best I can to find the right people, provide references, and answer quickly when researchers have requests. I guess I could do more, like anyone ... but well …I try to answer everything you ask quickly and as best I can”* [KU2].

From document analysis, it was also found that there was no rule about the role to play in the research team. The distribution task table was developed as a guide, and members of the team who completed it were not required to comply with their choice. Thus, they had the opportunity to get involved in other phases of the research than those for which they were previously registered, or not get involved at all. Thus, a researcher who had registered to help with recruitment was not finally able to do so due to several trips abroad.

### Success Factors of This Collaboration

Two factors related to “Common agenda” component of the CI framework emerged from data analysis: (1) Previous experiences with the topic of the research project strengthened interest; and (2) A partnership based on “a give and take” relationship.

#### Previous Experiences With the Topic of the Research Project Strengthened Interest

All participants saw interest in the collaboration related to previous experiences. They mentioned that they participated in this project because the topic of patient involvement in HTA was of interest to them. This interest has been strengthened by previous experiences on the topic of patient involvement, which enable them to make a valuable contribution to the project.

Verbatim transcripts and field notes analysis revealed that two researchers and a knowledge user of the team had worked together on a previous project aiming to introduce the patient perspective in HTA at the local level. In their previous project, researchers and knowledge users conducted a systematic review of patient involvement in HTA and developed a framework for guiding patient involvement in HTA at the local level.^31^ The new project built on the previous experience and aimed to use the framework developed in the previous phase to implement and then evaluate interventions involving patients in the assessment of alternatives to seclusion and restraint for hospitalized adults or those in long-term care facilities.^15^ In addition, three of the researchers had already worked on the topic of patient involvement in the areas of nursing, communication or medicine.

As highlighted by a knowledge user, the two groups worked together because both had something to contribute on the topic of patient involvement.

*“[…]. I think that no matter what, in the final analysis, we have to collaborate because we all have something to bring to the table. We all engage in the same sort of active listening as long as there is a project that concerns us all...”* [KU2].

#### A Partnership Based on “a Give and Take” Relationship

In return for their contribution, two researchers (R1 and R2) expected that their participation would be professionally beneficial.

*“When I get involved, I like to give as much time as I can to the project, because I’m going to get something for myself. It’ll help me. So if it is something concerning which I do not have the expertise, I will not give them anything, and it will not bring me anything, so I prefer to devote my time to other projects”* [R2].

In this sense, they viewed utility in the results of the study: they might publish on the topic, broaden their understanding, and/or incorporate the theme of patient involvement in their work.

*“[…] this is the project of X, but I’m interested enough ... Then the part that I use to present [in scientific meetings] is always related to patient involvement. [...]I even adapted the framework to my research. So I found the project very interesting since we worked within the framework. [...]”* [R2].

The input of their participation is also a concern for knowledge users. They view a great opportunity to engage patients for the first time as stakeholders and not as a data source as is usually the case in a HTA (KU1 and KU2).

*“But what is certain is that this very interesting project draws on patient involvement as a data source. ... In this case, people were questioned and were a data sources, but they were at the same time stakeholders of the process”* [KU1].

## Discussion

This study explored the structuration of the collaboration between researchers and knowledge users in a HTA project. Overall, it showed that several factors related to symbolic order, political/economic and legal institutions helped shape this partnership:

First, the semantic institution level revealed that the use of concepts related to each field makes communication difficult. Knowledge users talked about difficulties to understand researchers’ theories while researchers pointed out unfamiliarity with HTA approaches. The literature recognizes a need for greater patient engagement in HTA^15^ and the number of studies in the topic has increased in recent years. Guidance is still needed regarding both rationales and methods for patient engagement in HTA and technology coverage decisions.^32^ Some frameworks to guide decisions about whom to involve, through which mechanisms, and at what stages of the HTA process have been developed for implementation in HTA agencies.^33,34^ Knowing that the different vocabulary of researchers and knowledge users is recognized as impeding the ability of each to understand the other,^35^ it is, therefore, important to develop a common language to support patient engagement efforts.^34^

Secondly, the results related to the political institutions show that using their expertise allows knowledge users to make a unique contribution to the various stages of the research. This observation is different from the observations of Kothari^4^ who notes that researchers’ training and expertise give them more control over almost all stages of the research process compared to knowledge users who are mostly involved in disseminating or implementing the results. One can, therefore, argue with Vhonani^36^ that the adoption of an equity principle suggesting that each had something to offer in the relationship and therefore, no one should be more important than the other can help maintain a balance of power between researchers and knowledge users engaged in a HTA process.^36,37^

Finally, the lack of clear information on the specific role to play in the research is revealed through the legal institution. The researchers found that their expectations about knowledge users are not met, while the latter find they respond to requests from researchers. Clear and transparent expectations are recognized as a core principle for a successful partnership.^38^ However, this represents (with roles) an area that shows discrepancies between researchers and knowledge users.^37^ It should, therefore, be important to ensure a common understanding of expectations and roles and the contribution of each of these to foster trust and limit role confusion and misconceptions.^2,13^

## Study Limitations

As this project encompassed her thesis field, the author was in close contact with some members of the research team, which could have influenced her interpretation of the results. However, field notes compiled in a diary, documenting impressions and helping identify possible influences, mitigated this bias. Since the sample was small and data did not reach saturation, the results should be taken with a degree of caution. Moreover, participants may have been reluctant to criticize other members of the research team, causing a social desirability bias. Despite these limitations, the strength of this exploratory pilot study is that it could serve as a solid first step for a longer, broader, and deeper final study of the topic.

## Conclusion

Beyond the simple observation of problems that hinder collaboration between researchers and knowledge users and reading of factors facilitating and constraining this collaboration, the experience reported here explains the impact of social context on this process. Thus, the study opens the door to a poorly documented field in the area of HTA. Additional studies could build on these early observations and should address important questions such as who are the most appropriate stakeholders for the collaboration at different stages of the HTA process, and how they should be involved to most effectively inform the process.

## Acknowledgments

I am very grateful to Professor Marie-Pierre Gagnon who read and commented upon the first drafts of this paper. I also want to acknowledge the contribution of Marie Desmartis for data analysis. Many thanks to all key informants who participated in the study. A special thank to the four anonymous reviewers for their thoughtful suggestions that helped improving this paper. Thank you to Anne McBryde and Marcia Symphorien Laurent for editing the translated manuscript.

## Ethical issues

The Ethics Research Committee of the CHU de Québec approved the research project of which this study is a part (Approval # S11-06-039), and all participants but one, whose consent was verbal, signed a consent form. Before interviews, the author reminded participants of her role as a student and the importance of their input as regards understanding collaboration between researchers and knowledge users. Interviews were recorded with the consent of respondents. The author also coded interviews to ensure the anonymity of each participant.

## Competing interests

Author declares that she has no competing interests.

## Author’s contribution

MTD is the single author of the paper.

## Key messages

**Implications for policy makers**

- The use of concepts related to each field, the use of related expertise, and a lack of clearly defined roles in the project help shape the collaboration between researchers and knowledge users. Policy-makers may consider these issues when calling for more interaction between researchers and knowledge users.
- An office design allowing informal meetings (outside of team meetings) between team members working in the same building versus those located elsewhere contribute to unequal access to the information. Strategies are needed to enhance frequent informal meetings and a better flow of information between researchers and knowledge users working on a collaborative project.

**Implications for public**

Collaboration between researchers and knowledge users is seen as a strategy to enhance the effective translation of research results into actions to improve population well-being. However, little is known about this process when adopting a health technology. We looked at the practices of the actors involved and how, through them, they build and maintain this relationship in a health technology assessment (HTA) research project. We found that the use of concepts related to each field, the use of their expertise, and a lack of clearly defined roles in the project structured the collaboration between researchers and knowledge users. Given the increased call for patient involvement in HTA, such partnerships could indeed facilitate this process, as it usually requires a qualitative approach, grounded in social sciences, that knowledge users are not familiar with. Therefore, additional studies that build on these observations are needed to advance knowledge on this topic in the field of HTA.
